# Targeted RNA‐sequencing assays: a step forward compared to FISH and IHC techniques?

**DOI:** 10.1002/cam4.2599

**Published:** 2019-10-25

**Authors:** Gaëlle Tachon, Ulrich Cortes, Sophie Richard, Sébastien Martin, Serge Milin, Camille Evrard, Corinne Lamour, Lucie Karayan‐Tapon

**Affiliations:** ^1^ Laboratoire de Neurosciences Expérimentales et Cliniques Inserm U1084 Poitiers France; ^2^ Université de Poitiers Poitiers France; ^3^ Laboratoire de Cancérologie Biologique CHU de Poitiers Poitiers France; ^4^ Service d'Anatomo‐Cytopathologie CHU de Poitiers Poitiers France; ^5^ Service d'Oncologie CHU de Poitiers Poitiers France

**Keywords:** cancer genetics, lung cancer, medical genetics, next‐generation sequencing

## Abstract

**Introduction:**

*ALK* and *ROS1* rearrangements are molecular targets of several tyrosine kinase inhibitors. RNA‐sequencing approaches are regarded as the new standard for fusion gene detection, representing an alternative to standard immunohistochemistry (IHC) and fluorescence in situ hybridization (FISH) techniques.

**Patients and Methods:**

We aimed to compare two recent amplicon‐based RNA‐sequencing techniques: FusionPlex^®^ Alk Ret Ros1 v2 Kit (Archer^®^) with FHS‐003Z‐12—Human Lung Cancer Panel (Qiagen^®^) and assessed the accuracy of the data for therapy management. Thirty‐seven formalin‐fixed paraffin‐embedded non‐small cell carcinoma (NSCC) lesions initially explored by IHC and FISH were selected for RNA‐sequencing analysis.

**Results:**

Qiagen^®^ and Archer^®^ kits produced similar results and correctly identified 85.1% (23/27) and 81.5% (22/27) of IHC/FISH *ALK*‐ and *ROS1*‐positive samples, respectively, and 100% (6/6) of the negative samples. With regard to the ambiguous IHC‐positive/FISH‐negative cases, RNA‐sequencing confirmed 75% (3/4) of the FISH conclusion. Although not statistically significant, patients with common *EML4‐ALK* variants presented shorter overall survival and progression‐free survival compared with patients harboring rare variants.

**Conclusion:**

Our findings assessed the implementation of RNA‐sequencing approaches to explore *ALK* and *ROS1* rearrangements from formalin‐fixed paraffin‐embedded samples. We highlighted the similarities between Qiagen^®^ and Archer^®^ kits in terms of handling time, cost, and outcomes. We confirmed the feasibility of molecular testing in routine organization and its possible use not only as an alternative for standard IHC and FISH techniques, but as a supplementary technique helping to classify discrepant cases.

## Graphical abstract

To explore different fusion genes in a single assay and to detect gene partners engaged in *ALK* (anaplastic lymphoma kinase) or *ROS1* fusion, we evaluated two commercially available targeted RNA‐sequencing assays in a set of 37 tumor samples. The results of this study showed high concordance between targeted RNA‐sequencing and standard fluorescence in situ hybridization/immunohistochemistry (FISH/IHC) methods and illustrate the benefits of molecular technique to address the issue of FISH/IHC discordant cases.

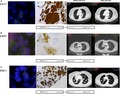

## 1 INTRODUCTION

ROS proto‐oncogene 1, receptor tyrosine kinase (*ROS 1*), and anaplastic lymphoma kinase (*ALK*) rearrangements are present in approximately 2% and 5% of non‐small cell lung cancers (NSCLCs), respectively. Fusion proteins resulting from these chromosomal rearrangements harbor strong oncogenic properties^1^ and are prime targets in cancer therapeutics. In this context, ALK tyrosine kinase inhibitors, such as crizotinib, were designed and approved by the FDA in 2011 for *ALK*‐rearranged NSCLCs. In the same way, NSCLC patients with *ROS1* rearrangements may benefit from crizotinib since 2016. Among these rearrangements involving different partners, *EML4* is the most frequent *ALK* partner (77%)^2^ but several others have also been described.^3^ As over‐activation of ALK tyrosine kinase or ROS1 tyrosine kinase is a prerequisite oncogenic event for cell transformation, the identification of fusion partners is not needed in kinase inhibitor therapy and is therefore rarely or never carried out. The standard methods currently used (fluorescence in situ hybridization [FISH] and immunohistochemistry [IHC]) to evaluate *ROS1* and *ALK* rearrangement do not provide information about gene partners and the clinical significance of accurate gene fusions remains unclear.^4^

IHC is a technique widely implemented in routine pathology laboratories and has proved to be an interesting prescreening test, which is inexpensive and easy to use.^5^ However, IHC is a targeted technique exploring ALK and ROS1 separately, therefore requiring a double amount of tumor material. In addition, IHC interpretation remains difficult, time‐consuming in comparison with RNA‐seq techniques, and requires the skills of a trained pathologist.^6^ Indeed, as long as the bioinformatic pipeline is well‐configured, the RNA‐seq will give a twofold response: presence or absence of gene fusion. On the contrary, IHC is not a binary test as positivity depends on the percentage of tumor cells stained and the intensity of the staining; it therefore requires more time for interpretation. The IASLC guidelines recommend IHC as the screening method for selecting specimens before FISH testing.^7^

The admitted gold standard assay for detection of *ALK* and *ROS1* rearrangements is the FISH technique using dual‐labeled break‐apart probes.^7^ Therefore, large amounts of tumor material must be available for both the IHC pres‐screening test and the subsequent FISH testing. Comparative studies have reported high but not fully equivalent concordance rates between the two techniques.^8^ Strikingly, positive IHC cases have been reported without molecular rearrangement by FISH, and conversely. Such ambiguous cases are a challenge for therapeutic decisions.

Molecular approaches could be useful as a means of ascertaining discordant and ambiguous cases.^9^, ^10^ Indeed, targeted RNA‐sequencing (targeted RNA‐seq) can achieve thorough detection and molecular characterization of several gene rearrangements concurrently, notably *ALK* fusions, and also *ROS1* or *NTRK* fusions. Next‐generation (NGS) targeted RNA‐sequencing technology, with gene‐specific primers designed in combination with universal primers, enables detection of any fused partner without “a priori” knowledge.^11^ Such information may have a predictive value for responses to targeted therapies.^4^ Several targeted RNA‐seq assays have reached the market, with reliable results, but no comparative testing has been performed. In addition, unlike *ALK* rearrangement, detection of *ROS1* rearrangement has never been fully assessed using latest generation assays.

To address these topics, we evaluated two targeted RNA‐seq, the FusionPlex^®^ Alk Ret Ros1 v2 Kit (Archer^®^) and the FHS‐003Z‐12—Human Lung Cancer Panel (Qiagen^®^). We aimed to determine the relevancy of these two methods for routine practice and to assess whether RNA‐seq technology will ensure correct and reliable information for therapy management.

## 2 MATERIALS AND METHODS

### 2.1 Patients and samples

Forty‐one NSCLC samples were selected based on routine molecular test results obtained at the Cancer Biology Department of Poitiers University Hospital between April 2014 and November 2017. The study was performed in accordance with French legislation (DC‐2015‐2449) and with the Declaration of Helsinki. Privacy of the data was ensured for all patients.

Among the 41 samples, four (9.8%) were excluded due to an insufficient amount of available RNA. Clinical data of the 37 selected patients include age, gender, smoking status, tumor stage, and sites, and are displayed in Table 1. Tumor samples were fixed in 4% formalin and embedded in paraffin (FFPE) according to standard procedure after surgical biopsy. Percentage of malignant cells was determined by trained pathologists of the university hospital. Histological data of the samples are available in Table S2.

**Table 1:** Clinical and histological patient characteristics

Characteristics	n	%
Age on diagnostic		
<65	14	38
≥65	23	62
Median	65 (33‐81)	
Gender		
Male	16	43
Female	21	57
Smoking status		
Have smoked	11	30
Smoked	6	16
Nonsmoker	9	24
Unknown	11	30
Type of specimen		
Biopsy	23	62
Surgical specimen	11	30
Core‐biopsy	2	5
Cytology	1	3
Site		
Lung	22	59
Lymph node	7	19
Other	8	22
Tumor stage		
T1	6	16.2
T2	4	10.8
T4	20	54.1
Unknown	7	18.9
WHO		
0‐1	24	65
2	1	3
3	3	8
4	2	5
Unknown	7	19
Treatment		
Surgery		
Yes	10	27
No	21	57
Unknown	6	16
Radiotherapy		
Yes	10	27
No	19	51
Unknown	7	19
Chemotherapy		
Yes	25	68
No	5	14
Unknown	7	19
Targeted therapy (among *ALK/ROS1* pos, n = 27)		
Yes (crizotinib or ceritinb)	15	56
No	7	26
Unknown	5	19

Abbreviation: pos, positive. WHO, World Health Organization

Three control samples, HD784, HD796, and HD783, were purchased from Horizon Discovery, harboring well‐characterized fusion transcripts (positive control) or no fusion (negative control). Two SureShot^™^ control samples from Archer^®^ were also tested, harboring *ROS1* rearrangement or no fusion, respectively.

### 2.2 IHC testing

Samples were screened for ALK rearrangements by IHC staining using the D5F3 monoclonal antibody and, according to the Ventana protocol, adjusted to the laboratory constraints. ROS1 IHC was performed using prediluted D4D6 monoclonal antibody in accordance with Genemed instructions. The percentage of ALK‐ or ROS1‐positive cells was evaluated by trained pathologists as part of routine testing and scored as follows: score 0, no staining; 1+, faint cytoplasmic staining; 2+, moderate, smooth cytoplasmic staining; or 3+, intense granular cytoplasmic staining. All samples with scores of 1+, 2+, or 3+ for cytoplasmic staining in more than 10% of tumor cells were considered ALK‐positive.^12^ All samples with cytoplasmic and membrane staining intensity of 1+, 2+, or 3+ were defined as ROS1‐positive and were automatically directed to FISH screening, regardless of the % of stained cells.^13^

### 2.3 FISH testing

Vysis *ALK* Break Apart FISH probes^©^ (Abbott Molecular) and ZytoLight^®^ *ROS1* SPEC Dual Color Break Apart were used for the detection of *ALK* and *ROS1* rearrangements, respectively. Zeiss Axio Imager Z2 fluorescent microscope and ISIS software^©^ (Meta System) were used for image acquisition. FISH results were evaluated by trained molecular biologists as part of routine molecular testing. Samples were considered *ALK*‐positive if ≥15% of tumor cells showed isolated red signal(s) and/or split red and green signals.^14^ Polysomy was defined by an average copy number of *ALK* signal ≥6.^15^ FISH‐positive cases for *ROS1* rearrangements were defined by more than 15% split or single green signals.^16^ Evaluation was performed in preselected areas rich in tumor cells, on at least 50 tumor cells. Ambiguous cases were checked a second time and an additional count of 50 cells was carried out.

### 2.4 RNA extraction

RNA was extracted from 1 to 4 FFPE sections of 10 µm using Maxwell 16 LEV RNA FFPE extraction kit according to manufacturer's instructions. RNA samples were then quantified using QuantiFluor^®^ RNA System on a Quantus^™^ Fluorometer (Promega).

### 2.5 Targeted NGS and data analysis

The Human Lung Cancer Panel kit (Qiagen) was designed to detect multiple gene rearrangements, among which *ALK* and *ROS1* are major targets. This RNA‐seq assay uses single primer extension and unique molecular barcodes to overcome traditional RNA‐seq limitation and to ensure increased precision and accuracy. Briefly, a minimum of 20 ng of RNA was initially converted into a first cDNA strand, followed by second‐strand synthesis to generate double‐stranded cDNA (ds‐cDNA). The ds‐cDNA was then end‐repaired, A‐tailed, and ligated at 5′ end to a specific adapter containing unique molecular barcode and sample index. Single primer extension was then performed to ensure target enrichment. Universal PCR amplification was then carried out followed by the addition of a second sample index to achieve dual indexing.

Libraries were then quantified with a Quantus^™^ Fluorometer (Promega), pooled at equimolar concentrations, and sequenced on a Miseq sequencer (Illumina). Sequencing data were analyzed using QIAseq Targeted RNAscan Panel Analysis Software 2.0. Only high confidence fusion calls were taken into account in this study.

FusionPlex^®^ Alk Ret Ros1 v2 (Archer) was designed to exclusively detect *ALK*,* RET*, and *ROS1* rearrangements with any other gene partner. A minimum of 50‐ng RNA was used as input according to the manufacturer's recommendations. The experiment was carried out as previously described.^11^ In brief, cDNA was first synthesized from the RNA using random priming, then end‐repaired, followed by dA‐tailing, and adapter ligation. Molecular barcoding and sample barcoding were both incorporated during FusionPlex library ligation. Two rounds of PCR amplification with gene‐specific primers were then carried out. Lastly, libraries were quantified, pooled at equimolar concentration, and sequenced as described above. The fastQ files generated were analyzed using Archer analysis software v5.1. Only strong evidence fusions were taken into account for fusion calling.

Both assays were designed to detect fusions without “a priori” knowledge of the partner.

### 2.6 Sanger sequencing

Exploration of *ALK* rearrangements in discordant cases was assessed directly by Sanger sequencing on sample libraries generated from Qiagen or Archer kits. Nested PCR was performed using the following primers: Forward P5: 5′‐AATGATACGGCGACCACCGA‐3′ (P5 region of adapter construct) with Reverse ALK20.1rc: 5′‐CCTGGTGCTTCCGGCGGTACA‐3′ (*ALK* exon 20) for the first PCR and P5 5′‐AATGATACGGCGACCACCGA‐3′ with ALK20.2rc: 5′‐CCATCTGCATGGCTTGCAGCT‐3′ for the nested PCR. Both PCR amplifications were performed as follows: 95°C for 15 minutes, 40 cycles of denaturation at 95°C for 20 seconds; annealing at 53°C for 30 seconds; extension at 72°C for 20 seconds, and a final extension at 72°C for 5 minutes. Samples were sequenced with a 3500Dx DNA Sequencer (Applied Biosystems).

## 3 RESULTS

### 3.1 IHC and FISH testing

Among 37 samples, 24 were considered *ALK*‐positive and three samples were *ROS1*‐positive based on at least IHC signals of 1+ associated with positive FISH results. Six *ALK/ROS1*‐negative samples presented IHC scores from 0 to 1+ without *ALK/ROS1* rearrangement detected by FISH. Finally, four ambiguous samples were defined as significant IHC signals (from 2+ to 3+) and with FISH‐negative results. These results are summarized in Table 2.

**Table 2:** Summary of *RNA*‐sequencing outcome of 37 tumors

	Sample ID	IHC		FISH	RNA‐seq results			Concordance
		ALK	ROS1	FISH	*ALK/ROS1* rearrangement	Transcript	Variant	Qiagen/Archer
Positive cases	E3837	3+		*ALK* pos	*EML4‐ALK*	E6:A20	V3a/b	Yes
	E3959	3+		*ALK* pos	*EML4‐ALK*	E13:A20	V1	Yes
	E4428	3+		*ALK* pos	*EML4‐ALK*	E13:A20	V1	Yes
	E4513	2+		*ALK* pos	*EML4‐ALK*	E13:A20	V1	Yes
	E4559	2+		*ALK* pos	*EML4‐ALK*	E6:A20	V3a/b	Yes
	E4883	2+		*ALK* pos	*EML4‐ALK*	E6:A18	Unknown	Yes
	E4974	3+		*ALK* pos	*EML4‐ALK*	E13:A20	V1	Yes
	E4169	3+		*ALK* pos	*EML4‐ALK*	E6:A20	V3a/b	Yes
	E4380	2+		*ALK* pos	*EML4‐ALK*	E13:A20	V1	Yes
	E2895	2+		*ALK* pos	*EML4‐ALK*	E13:A20	V1	Yes
	E2840	2+		*ALK* pos	*EML4‐ALK*	E2:A20	V5	Yes
	E3003	3+		*ALK* pos	*EML4‐ALK*	E18:A20	Unknown	Yes
	E3188	3+		*ALK* pos	*EML4‐ALK*	E2:A20	V5	Yes
	E3185	3+		*ALK* pos	*EML4‐ALK*	E6:A20	V3a/b	Yes
	E3551	3+		*ALK* pos	*EML4‐ALK*	E20:A20	V2	No (Archer NA)
	E2054	2+		*ALK* pos	*EML4‐ALK*	E6:A20	V3a/b	Yes
	E4721	3+		*ALK* pos	*DCTN1‐ALK*	E26:A20	Rare	Yes
	E3040	3+		*ALK* pos	*DCTN1‐ALK*	E26:A20	Rare	Yes
	E3252	3+		*ALK* pos	*DCTN1‐ALK*	E26:A20	Rare	Yes
	E5011	3+		*ALK* pos	*HIP1‐ALK*	E28:A20	Rare	Yes
	E4115		2+	*ROS1* pos	*CD74‐ROS1*	E6:E34	—	Yes
	E4268		3+	*ROS1* pos	*CD74‐ROS1*	E6:E34	—	Yes
	E4610		2+	*ROS1* pos	*SLC34A2‐ROS1*	E4:E32	—	Yes
	E4349	3+		*ALK* pos	NEG	—	—	Yes
	E3276	2+		*ALK* pos	NEG	—	—	Yes
	E2333	1+		*ALK* pos	NEG	—	—	Yes
	E4170	3+		*ALK* pos	NEG	—	—	Yes
Ambiguous cases	E5693	3+		*ALK* neg	*EML4‐ALK*	E13:A20	V1	Yes
	E5046	3+		Polysomy	NEG	—	—	Yes
	E5193	2+		*ALK* neg	NEG	—	—	Yes
	E5680	3+		*ALK* neg	NEG	—	—	Yes
Negative cases	E4378		1+	*ROS1* neg	NEG	—	—	Yes
	E4598	1+		*ALK* neg	NEG	—	—	Yes
	E4634	1+		*ALK* neg	NEG	—	—	Yes
	E4674	NEG		*ALK* neg	NEG	—	—	Yes
	E4720		1+	*ROS1* neg	NEG	—	—	Yes
	E5844	1+		*ALK* Atypical pattern	NEG	—	—	Yes

Abbreviations: FISH, fluorescence in situ hybridization; ID, identity; IHC, immunohistochemistry; NA, not amplified; neg, negative; pos, positive.

### 3.2 RNA‐sequencing results obtained with reference samples

Three positive (HD784, HD796, and SureShot‐ROS1) and two negative (HD783 and SureShot‐Neg) controls, fully characterized and of high quality, were used to assess performance of the two methods. SureShot controls are RNA samples while HD784 and HD796 controls are cell lines‐derived FFPE sections extracted and analyzed under the same conditions as patient samples, thereby ensuring the validity of the experiment from the extraction step.

HD784 harbors *EML4‐ALK, CCDC6‐RET*, and *SLC34A2‐ROS1* fusions. HD796 further contains *TPM3‐NTRK1* and *ETV6‐NTRK3* fusions. SureShot‐ROS1 harbors *SLC34A2‐ROS1* fusion. HD783 and SureShot‐Neg are negative controls containing none of the previous fusions.

Archer and Qiagen fusion assays successfully identified *EML4‐ALK, CCDC6‐RET*, and *SLC34A2‐ROS* *1* fusions. *TPM3‐NTRK1* fusion was detected only by Qiagen assay as the specific Archer FusionPlex^®^ assay tested was not designed for NTRK1 detection. Neither of the two assays was designed for *ETV6‐NTRK3* detection. No false‐positive was reported by either assay, thereby confirming their specificity (Table S1). To evaluate the robustness of both approaches, HD784‐positive control was performed twice in two independent runs with freeze‐thaw cycles of RNA. *EML4‐ALK*, *CCDC6‐RET*, and *SLC34A2‐ROS1* fusions were properly called in all cases with a high confidence index.

### 3.3 RNA‐sequencing results on characterized clinical samples

Among the 41 patients initially selected, 9.8% (4/41) could not be analyzed because of the insufficient amount of RNA obtained after extraction (<20 ng). Samples defined as positive according to IHC/FISH methods were correctly identified by the Qiagen fusion assay with 85% concordance (23/27) and by the Archer fusion assay with 82% concordance (22/27). No fusion transcripts were incorrectly called by either method (Table 2). The only discrepancy between the two assays involved one *ALK* rearrangement undetected by the Archer assay; however, this sample did not fulfill the quality assessment of the manufacturer (Ct > 30) and therefore would not have been explored under clinical practice. Quality assessment of original samples appears to be more restricted for the Archer assay in comparison with the Qiagen assay.

Most fusions detected were commonly described fusions with 15 *EML4‐ALK* (six E13:A20, six E6:A20, two E2:A20, and one E20:A20), one *SLC34A2‐ROS1*, and two *CD74*‐*ROS1* variants. Interestingly, several infrequent transcripts were also detected, three samples showing *DCTN1‐ALK*, one sample with *HIP1‐ALK,* and two samples with rare *EML4‐ALK* fusions (E6:A18 and E18:A20). All of these fusion transcripts were analyzed by both Qiagen and Archer analysis software (Table 2).

No fusion transcripts were reported for *ALK*‐negative samples (E5844, E4598, E4634) or for *ROS1*‐negative samples (E4378, E4720, E4674), nor were *ALK* fusions observed in *ROS1*‐positive samples.

Four samples presented relevant IHC signals for ALK (2+ or 3+), although showing FISH‐negative results (Table 2). In one sample (E5693), the IHC result was confirmed by both RNA‐seq assays (25%, 1/4), in the other three samples (E5046, E5193, E5680) RNA‐seq assays contradicted IHC results, thereby corroborating FISH conclusions (75%, 3/4). Consequently, therapeutic approaches were adjusted for patient E5693 according to RNA‐seq findings. Among these ambiguous cases, it is worth noting that the E5046 strong IHC signal (3+) was correlated to *ALK* polysomy phenotype, disclosed by FISH. Significant ALK IHC staining of E5193 was established on 100 cells only (25% of tumor cells in the sample) and patient E5680 was an active smoker with large cell neuroendocrine carcinoma, not prone to present* ALK* rearrangement.

**Figure 1:**
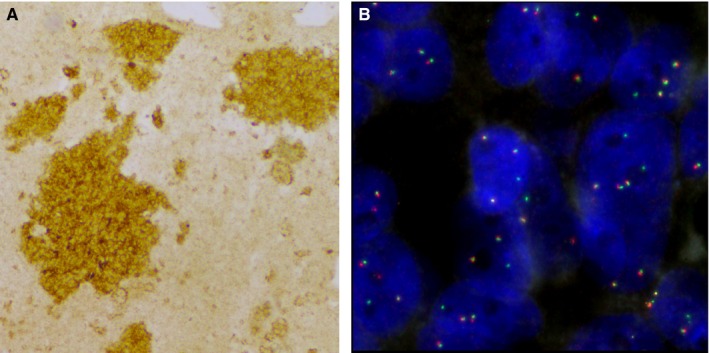
IHC and FISH analyses of *ALK* rearrangement of E5844 tumor. A, Photograph of IHC cytoplasmic staining in more than 10% of tumor cells at a score of 1+ (×10). B, Photograph of FISH showing atypical pattern of fused signal associated with isolated green signal (×100). FISH, fluorescence in situ hybridization; IHC, immunohistochemistry

Another case (E5844), with faint IHC staining presented an unusual *ALK* FISH pattern with one fusion and one deleted (green) signal in most nuclei (Figure 1). In this case, no fusion was detected by RNA‐seq assays. Therefore, RNA‐seq assays are the most suitable techniques to explore such ambiguous cases as these assays are designed to detect any *ALK* or *ROS1* fusion partner.

Four samples (E4349, E3276, E2333, E4170) showed discordant results despite FISH/IHC positive records (4/27, 14.8%), both RNA‐seq assays indicating the absence of gene fusion (Table 2). In three samples (E4170, E2333, E4349), the amount of residual material allowed us to perform nested PCR on library products followed by Sanger sequencing analysis (Figure S1). All three patients showed *EML4‐ALK* fusion transcripts, specifically variants 3 and 5 in two cases, whereas the fusion point was difficult to establish precisely in the third patient. Noticeably, E4349 and E4170 samples presented a low percentage of tumor cells (20%) and only a small amount of RNA had been extracted and was available for RNA‐seq analysis (3.1 and 7.2 ng/µL, respectively), which may explain the false‐negative result (Table S2). IHC of E2333 was performed despite a limited percentage of tumor cells (15%) and FISH positivity was set at the reference threshold of 15%. Therefore, it may be possible that remaining material for RNA‐seq analysis contained less than 15% of tumor cells, which hampered efficient detection of rearrangements. The same postulate can be made for E3276, with only 30% tumor cells explored by FISH analysis and the positivity established at the reference threshold.

### 3.4 Clinical relevance of rare fusion transcripts

Clinical data as well as progression‐free survival and overall survival were available for 29 patients. Among them, 18 presented *ALK* rearrangements with an identified partner revealed by RNA‐seq technology. Rare variants, with median overall survival of 66.6 months, appeared to be of better prognosis than common variants, namely variants 1 and 3, with median OS of 17.6 months, although these results did not reach statistical significance (*P* = .34) due to the reduced size of the cohort (Figure S2).

**Figure 2:**
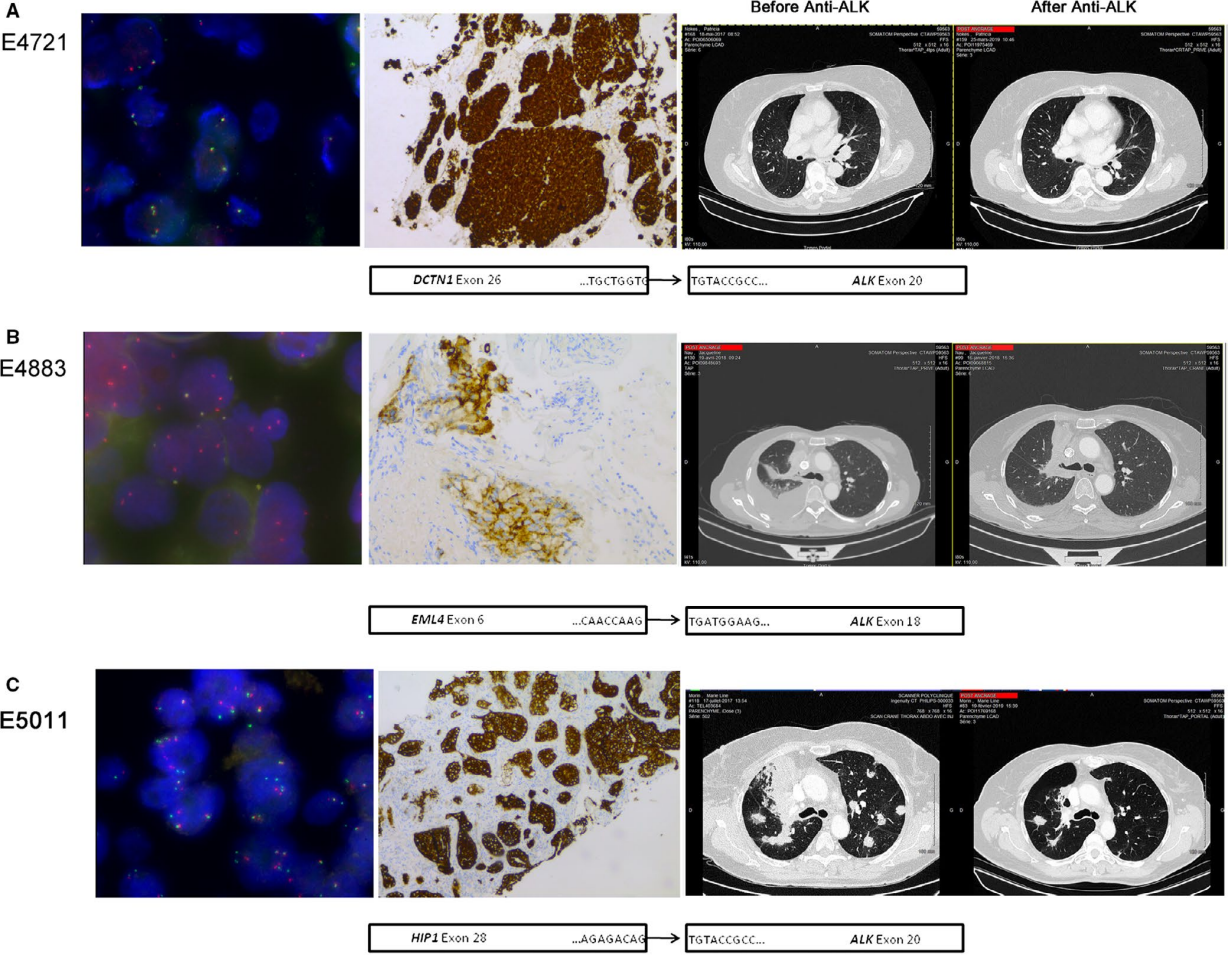
Representative images showing the presence of *ALK* rearrangements in E4721 (A), E4883 (B), and E5011 (C) patients. All left panels show representative images of rearranged tumors with either a classic break‐apart pattern with one fusion signal and two split red and green signals or a less common pattern with isolated red signal(s) combined with fused signals (×100). Representative medium (2+) to strong (3+) IHC staining are shown in middle panels. Thoracic computed tomography scans of patients before and after targeted therapy are shown in the right panels and schematic representations of the fusion transcript for each case are presented below the three panels. IHC, immunohistochemistry

For instance, patient E4721 with a rare gene fusion *DCTN1‐ALK* (E26:A20) received crizotinib at a dose of 250 mg twice a day as first‐line therapy. A major response was observed by computed tomography scanning 3 months after initiation of therapy evidenced by a 60% decrease in tumor size and stabilization of bone lesions (Figure 2A). The overall condition of the patient significantly improved with no major side effects. Since then, the patient has been treated with crizotinib (18 months thus far). Patient E4883 presented a specific *EML4‐ALK* rearrangement with breakpoint of the *ALK* gene occurring ahead of exon 18, (instead of exon 20, as usually described), preserving the tyrosine kinase domain. This patient received ceritinib, 5×150 mg, as second‐line therapy after a first‐line of crizotinib was discontinued due to hepatotoxicity. A dramatic response to targeted therapy was observed with major regression of the primary lung lesions and liver metastases (Figure 2B).

Finally, patient E5011 presented *HIP1‐ALK* rearranged adenocarcinoma with metastases spreading to the lung, meningitis, and pericardium at diagnosis. This patient refused encephalic radiotherapy but accepted targeted therapy based on crizotinib orally at a dose of 250 mg twice daily. The progression of the disease was stopped and lesions have been stabilized for now (13 months thus far) (Figure 2C).

### 3.5 Technical comparison of existing methods

IHC is easily automatizable, does not require additional steps such as RNA extraction, and requires a limited amount of tumor material (two FFPE sections of 4 µmol/L). The method is cost‐friendly and time‐saving even though two independent experiments have to be run (one for each protein explored). However, IHC provides overall information on protein expression but not on the mechanisms involved (gene amplification, rearrangement). In addition, interpretation of results may be difficult due to semiquantitative scoring of the signal, which does not allow definition of a proper threshold between negative and positive samples (Table 3).

**Table 3:** Technical comparison of existing methods

Technology	IHC	FISH	Archer fusion plex	Qiagen fusion kit
Approach	Protein	DNA, fluorescence	RNA‐seq	RNA‐seq
Original material	Two slides 4‐µm thick	Two slides 4‐µm thick	10‐µm section	10‐µm section
Extraction step	Not necessary	Not necessary	20‐250 ng RNA	10‐250 ng RNA
Targets	Single protein	Single splitting molecular region	*ALK, ROS1, RET*	*ALK, ROS1, RET,* and 24 other genes
Detectable alteration	Protein overexpression	DNA break‐apart	Fusion transcript	Fusion transcript
Analysis software	Not necessary	Not necessary	Provided by the supplier	Provided by the supplier
Technical time (in our laboratory, real practice)	1 d	2 d	5 d (sequencing included)	5 d (sequencing included)
Estimated expense/sample (in our laboratory, human resources not included)	63 USD	89 USD	318 USD	362 USD

Abbreviations: d, days; FISH, fluorescence in situ hybridization; IHC, immunohistochemistry; seq, sequencing; USD, United States dollar.

Similarly, FISH can also be automated, does not require DNA extraction, and can be achieved with low input of tumor material. FISH provides the advantage of differentiating between gene rearrangements, polysomy, and amplification processes and therefore gives more concise information for therapy purposes. To that extent, although the clinical significance of *ALK* amplification and polysomy remains uncertain, these alterations have been reported to be involved in resistance to crizotinib. However, this method neither identifies gene partners nor provides information on chromosomal rearrangements.

FusionPlex^®^ Alk Ret Ros1 v2 (Archer) and Human Lung Cancer Panel (Qiagen) are very similar targeted RNA‐seq technologies from two different suppliers.^17^ Both assays can detect the accurate fusion transcripts and can explore several genetic rearrangements concurrently. While human Lung Cancer Panel investigates more genes than FusionPlex^®^ Alk Ret Ros1 v2 assay (27 vs 3), both suppliers provide customized services for extending panels of target genes. The main disadvantages of RNA‐seq assays compared to IHC and FISH are the extra cost and increased processing time for library preparation and sequencing.

## 4 DISCUSSION

Detection of *ALK* and *ROS1* rearrangements by IHC prescreening and FISH confirmation is the standard process for identifying patients with NSCLC eligible for treatment with tyrosine kinase inhibitors.^18^ FISH/IHC discordances are common in routine practice and decision‐making on treatment with targeted therapies remains complicated for these patients. Recent studies have suggested that IHC testing alone may provide a better estimate of crizotinib response than FISH.^19^, ^20^ An observational study from the European Society for Medical Oncology reported that IHC testing was an ideal, robust, and accurate primary procedure to determine access to targeted therapy as long as quality testing and validation assays are performed.^21^ In compliance with this, patients from our cohort with strong IHC staining (2+ or 3+) could have received targeted therapy without FISH exploration. However, 75% of these patients were actually negative according to FISH analysis and did not present any *ALK* rearrangement according to the two independent RNA‐seq assays. IHC results were confirmed by RNA‐seq assays for one patient only (1/4). Hence, this study suggests that RNA‐seq analysis could be of major interest in settling FISH/IHC discordances. These observations are consistent with previous studies. For example, Vollbrecht et al. investigated 18 unequivocal and 15 equivocal samples through RNA‐seq‐based analysis with discordant results between FISH and IHC and identified three false‐positive FISH samples.^22^ Pekar‐Zlotin et al investigated the IHC and FISH profiles of 51 lung adenocarcinomas and confirmed discrepancies by NGS analysis. The authors identified false‐negative FISH samples and concluded that *ALK* testing should initially be based on IHC and/or NGS‐based methods instead of FISH testing.^9^ Finally, Jang et al. conducted a similar study and found that *ALK* FISH results were false‐positive in three out of four FISH‐positive/IHC‐negative cases and confirmed that NGS approach was most effective in detection of *ALK* rearrangements.^23^

Therefore, molecular approaches seem to be more reliable than FISH in detection of fusion genes and may become the new gold standard. In our study, RNA‐seq assays failed to detect four rearranged samples, and three of them were confirmed by Sanger experiment (14.8%, 4/27). Both companies reported technical sensitivity of 90%‐95%, nevertheless established on internal control samples. Our study showed the sensitivity of these techniques on real case samples and in routine laboratory conditions, although the number of samples was limited. Recent publications have also confirmed the considerable but not complete correlation between IHC/FISH and RNA‐seq explorations. In a cohort of 53 samples, McLeer‐Florin et al. reported RNA‐seq sensitivity of 80%.^24^ Letovanec et al. investigated a cohort of 96 cases and while positivity was defined as IHC‐positive/FISH‐positive, NGS sensitivity and specificity were only 85.0% and 79.0%, respectively.^25^ Moreover, NGS failed to provide results in 19% (18/95) of samples. In our study, 9.8% could not be analyzed, mostly due to an insufficient amount of RNA obtained after extraction (<20 ng). One of the challenging issues of RNA‐seq assay is the need for a large amount of good quality RNA, which may be difficult to obtain from biopsy specimens. Most of the false‐negative cases of this study presented a low percentage of tumor cells, which might have impaired RNA‐seq testing. Thereupon, most studies, ours included, carried out routine techniques (eg, IHC, FISH, molecular exploration) before RNA‐seq analysis, thereby reducing available material.

Prior to implementation of targeted RNA‐seq in clinical use in our laboratory, we assessed two commercially available assays. With the exception of one sample of insufficient quality for Archer analysis, both assays provided exactly the same information for all samples. Partner genes were clearly identified by both methods without discordance. With regard to processing time and cost per sample, both assays are basically equivalent, although more expensive than IHC and FISH. Furthermore, both RNA‐seq assays provided information about partner genes and will be of increased therapeutic interest in the near future. In this regard, Li et al. reported that patients with variant 2 *EML4‐ALK* tumors had longer progression‐free survival than patients with other variants.^4^ In another study, *EML4‐ALK* variant 3 was shown to be of poorer prognosis than other variants.^24^ On the contrary, Yoshida et al. showed that tumors with *EML4‐ALK* variant 1 preferentially responded to crizotinib compared with other variants.^26^ In our study, no significant differences were observed in overall survival and progression‐free survival when comparing rare fusion genes or rare *EML4‐ALK* variants with most common variants. Although not significant, there was a clear trend toward longer OS and PFS for patients with rare *EML4‐ALK* variants. The clinical impact of the different fusion partners remains to be determined along with its implications for therapeutic management.

Lastly, despite the higher cost and processing time, simultaneous analysis of several fusion genes represents a major advantage of the NGS methods. A recent study has shown that patients with *NTRK1*‐rearranged NSCLCs can benefit from entrectinib‐based therapy.^27^ Recently, the FDA approved larotrectinib for solid tumors with *NTRK* gene fusions. Another study has shown the good response of *RET*‐rearranged adenocarcinoma to cabozantinib, a multi‐kinase inhibitor.^28^ While fusion genes are rare events in lung adenocarcinoma, their presence is an indicator of possible targeted therapy. Moreover, fusion genes have also been reported in invasive mucinous adenocarcinoma^29^ and squamous and small‐cell lung cancers,^30^ and their exploration may soon be required in therapeutic management.^31^ Molecular pathology laboratories have already started receiving requests for exploration of various new fusions, for example, *NTRK* in lung cancer, *FGFR2* in colorectal cancer, *BRAF* in pilocytic astrocytoma. Therefore, instead of developing, validating, and certifying new IHC/FISH tests for all new markers, it might be preferable to implement RNA‐seq testing to explore all of them concurrently. Moreover, most genomic companies offer customized solutions for the design of RNA‐seq panels. Therefore, if a new fusion marker of interest is revealed for cancer therapy or prognosis, it can easily be added into the existing panel.

## 5 CONCLUSION

In conclusion, while RNA‐seq tests for *ALK* and *ROS1* fusion detection were achievable and provided full information regarding the gene partner, sensitivity did not reach 100% and several samples could not be analyzed due to low RNA amount. QIAseq human lung cancer targeted RNAscan panel and FusionPlex^®^ Alk Ret Ros1 v2 required similar operating practices and provided similar results with FFPE samples. Currently, in the absence of requirements for partner‐gene information prior to targeted therapy, RNA‐seq assays might be restricted to settling discordant cases with equivocal IHC/FISH results.

## CONFLICT OF INTEREST

None declared.

## Data Availability

The data that support the findings of this study are available on request from the corresponding author.
