# Supplementary material for: Targeted RNA‐sequencing assays: a step forward compared to FISH and IHC techniques?
Source: Cancer Med. 2019 Oct 25;8(18):7556–66. doi: 10.1002/cam4.2599 (PMC6912030; doi:10.1002/cam4.2599)
Supplement: Supplementary file 1 [file CAM4-8-7556-s001.pptx]

## Slide 1
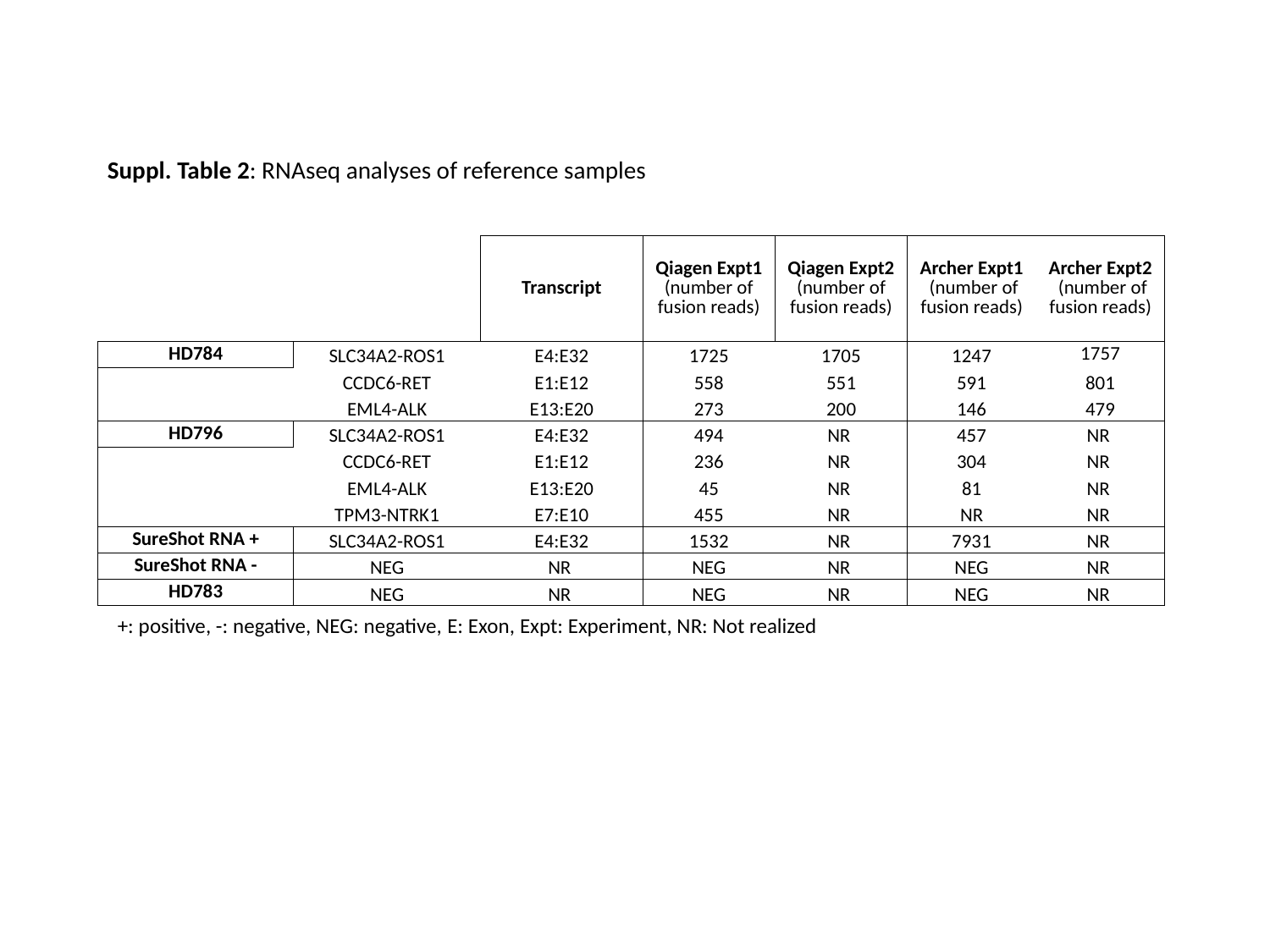

Suppl. Table 2: RNAseq analyses of reference samples
| | | Transcript | Qiagen Expt1 (number of fusion reads) | Qiagen Expt2 (number of fusion reads) | Archer Expt1 (number of fusion reads) | Archer Expt2 (number of fusion reads) |
| --- | --- | --- | --- | --- | --- | --- |
| HD784 | SLC34A2-ROS1 | E4:E32 | 1725 | 1705 | 1247 | 1757 |
| | CCDC6-RET | E1:E12 | 558 | 551 | 591 | 801 |
| | EML4-ALK | E13:E20 | 273 | 200 | 146 | 479 |
| HD796 | SLC34A2-ROS1 | E4:E32 | 494 | NR | 457 | NR |
| | CCDC6-RET | E1:E12 | 236 | NR | 304 | NR |
| | EML4-ALK | E13:E20 | 45 | NR | 81 | NR |
| | TPM3-NTRK1 | E7:E10 | 455 | NR | NR | NR |
| SureShot RNA + | SLC34A2-ROS1 | E4:E32 | 1532 | NR | 7931 | NR |
| SureShot RNA - | NEG | NR | NEG | NR | NEG | NR |
| HD783 | NEG | NR | NEG | NR | NEG | NR |
+: positive, -: negative, NEG: negative, E: Exon, Expt: Experiment, NR: Not realized

## Slide 2
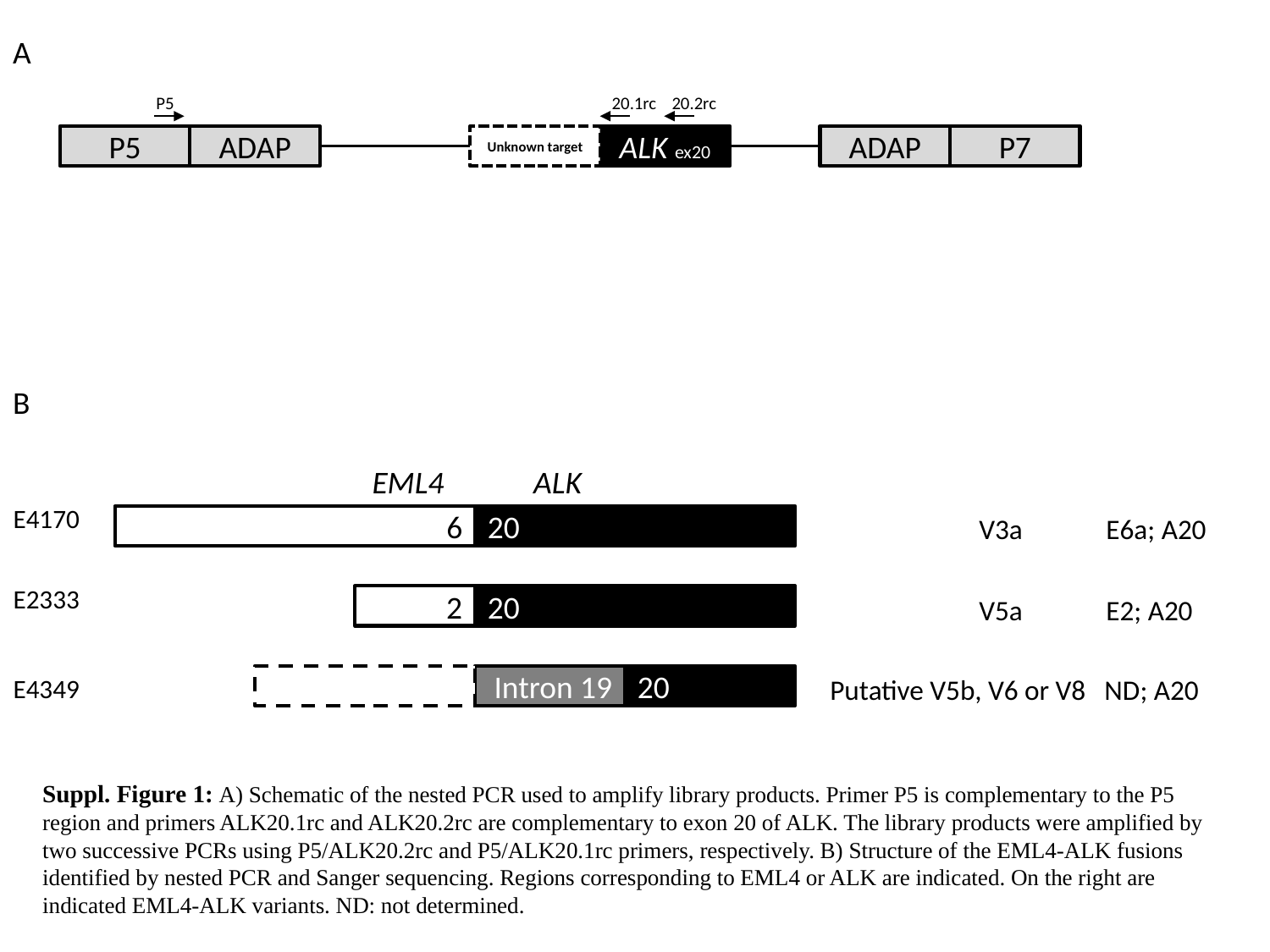

A
P5
20.1rc
20.2rc
P5
ADAP
Unknown target
ALK ex20
ADAP
P7
B
EML4
ALK
V3a	E6a; A20
6
20
2
20
V5a	E2; A20
Intron 19
20
Putative V5b, V6 or V8 ND; A20
E4170
E2333
E4349
Suppl. Figure 1: A) Schematic of the nested PCR used to amplify library products. Primer P5 is complementary to the P5 region and primers ALK20.1rc and ALK20.2rc are complementary to exon 20 of ALK. The library products were amplified by two successive PCRs using P5/ALK20.2rc and P5/ALK20.1rc primers, respectively. B) Structure of the EML4-ALK fusions identified by nested PCR and Sanger sequencing. Regions corresponding to EML4 or ALK are indicated. On the right are indicated EML4-ALK variants. ND: not determined.

## Slide 3
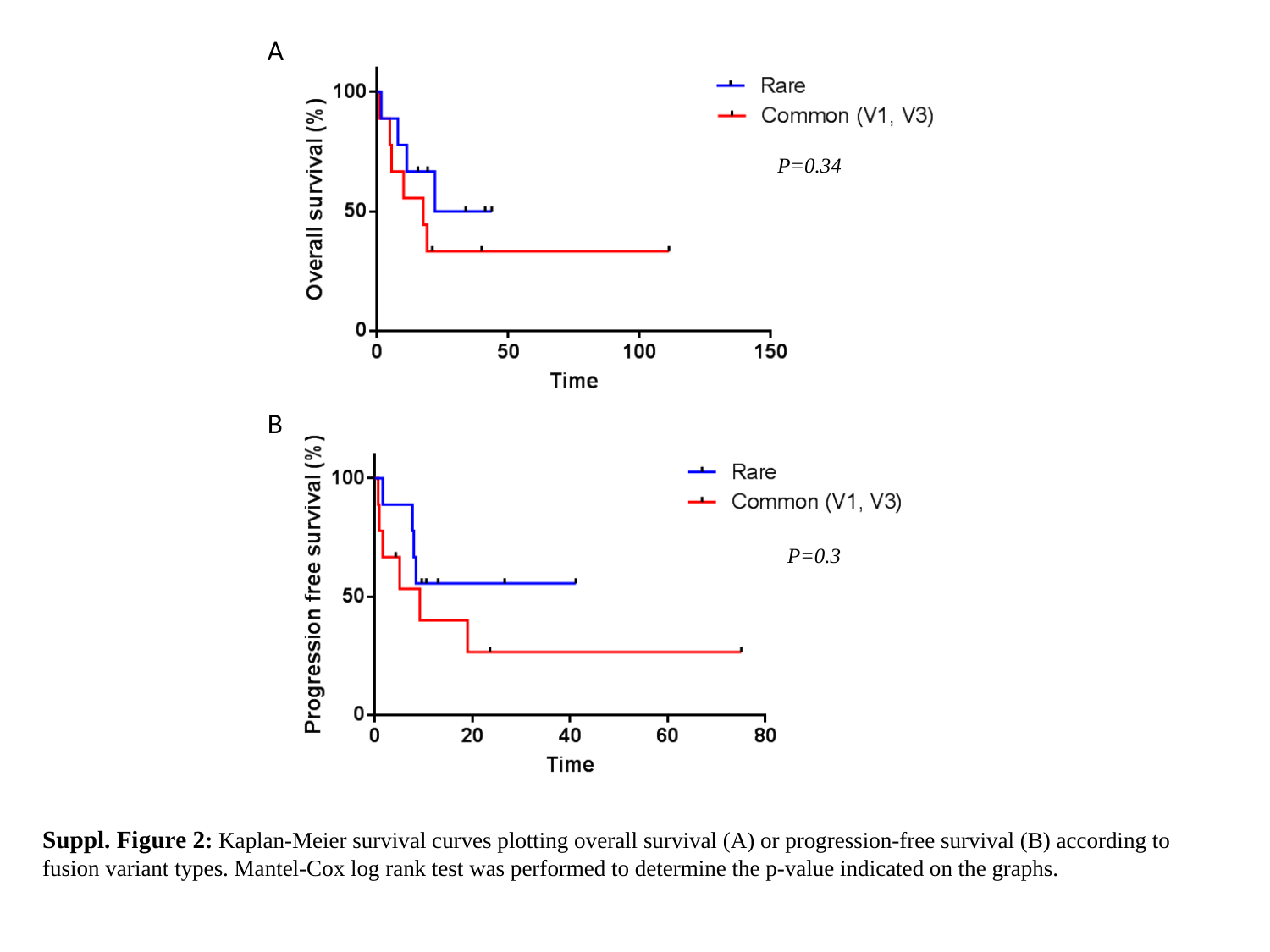

A
P=0.34
B
P=0.3
Suppl. Figure 2: Kaplan-Meier survival curves plotting overall survival (A) or progression-free survival (B) according to fusion variant types. Mantel-Cox log rank test was performed to determine the p-value indicated on the graphs.
